# Circulating miR-200c and miR-141 and outcomes in patients with breast cancer

**DOI:** 10.1186/s12885-015-1238-5

**Published:** 2015-04-02

**Authors:** Silvia Antolín, Lourdes Calvo, Moisés Blanco-Calvo, María Paz Santiago, María José Lorenzo-Patiño, Mar Haz-Conde, Isabel Santamarina, Angélica Figueroa, Luis Miguel Antón-Aparicio, Manuel Valladares-Ayerbes

**Affiliations:** 1 Medical Oncology Department, La Coruña University Hospital (CHUAC), Servicio Galego de Saúde (SERGAS), As Xubias, 84 PC 15006, La Coruña, Spain; 2 Translational Cancer Research Lab, Biomedical Research Institute (INIBIC), Carretera del Pasaje, s/n. PC 15006, La Coruña, Spain; 3 Pathology Department, La Coruña University Hospital (CHUAC), Servicio Galego de Saúde (SERGAS), As Xubias, 84 PC 15006, La Coruña, Spain; 4 Medicine Department, La Coruña University (UDC), Campus de Oza, s/n; PC 15006, La Coruña, Spain

**Keywords:** Breast neoplasm, microRNAs, Blood, Biomarkers, Prognostic factors

## Abstract

**Background:**

The deregulation of microRNAs in both tumours and blood has led to the search for microRNAs to indicate the presence of cancer and predict prognosis. We hypothesize the deregulation of miR-200c/miR-141 in the whole blood can identify breast cancer (BC), and could be developed into a prognostic signature.

**Methods:**

The expression of miR-200c and miR-141 were examined in bloods (57 stage I-IV BC patients and 20 age-matched controls) by quantitative reverse-transcription PCR. The associations of circulating microRNAs with clinic and pathological characteristics were analysed. Their effects on survival were analysed by the Kaplan-Meier method and Cox regressions.

**Results:**

MiR-200c was down regulated (*P* < 0.0001) in the blood of BC patients, yielded an area under the ROC curve of 0.79 (90% sensitivity, 70.2% specificity) in discriminating BC from controls. Circulating miR-141 was not discriminating. MiR-200c and miR-141 in the blood of BC patients were inversely correlated (*P* = 0.019). The miR-200c levels were numerically higher in stage IV and tumours with lower MIB-1. MiR-141 was significantly higher in the blood of patients with stage I-III, lymph node metastasis, and HER2 negative tumours. High blood expression of miR-200c and/or low expression of miR-141 was associated with unfavourable overall survival (hazard ratio, 3.89; [95% CI: 1.28-11.85]) and progression-free survival (3.79 [1.41–10.16]) independent of age, stage and hormonal receptors.

**Conclusions:**

Circulating miR-200c and miR-141 were deregulated in BC comparing with controls. Furthermore, miR-200c and miR-141 were independent prognostic factors and associated with distinct outcomes of BC patients.

**Electronic supplementary material:**

The online version of this article (doi:10.1186/s12885-015-1238-5) contains supplementary material, which is available to authorized users.

## Background

Breast cancer (BC) is the leading cause of cancer death in women worldwide, accounting for 458,400 deaths in 2008 [1]. Relative survival from BC in women has improved steadily in all developed countries over the past 25 years. By (2012), it was estimated that Spain would have a total of 27,000 new diagnoses of BC among women and currently BC remains the leading cause of death among women in Spain with 6231 deaths and a European age-standardised mortality rate of 18 per 100,000 person-years [2].

Cancer progression and blood-borne metastasis contribute to the great majority of BC deaths. The discovery of specific biomarkers characterizing the metastatic phenotype holds the promises of personalised therapy and improved prognosis prediction in many neoplastic diseases including BC.

Tumour tissue based biomarkers (e.g. size, grade, node status, hormone receptor status, HER2, Ki-67) are widely used in the clinical practice in BC. In addition, gene expression signatures of breast carcinomas have led to new classifications of tumour subgroups and also carry prognostic and predictive information [3]. In contrast, although serum tumour markers, including carcinoembryonic antigen, CA 15.3 and CA 27.59 could provide some prognostic information, they are not currently recommended for screening, diagnosis, or routine surveillance after initial treatment [4].

A large amount of data has revealed the correlation between specific tumours and differential microRNA (miRNA) expression profiles, thus providing a new class of disease-specific biomarkers (revised in [5]). MiRNAs are 18- to 25-nt noncoding RNA molecules that regulate protein expression of specific mRNA by either translational inhibition or mRNAs degradation. MiRNAs play different regulatory roles in cancer and have distinct functions in controlling the cell cycle, proliferation, invasion and metastasis. Moreover, miRNA deregulation can induce a pro-inflammatory and pro-metastatic environment and curtail the anti-tumour immunity [6,7].

An increasing number of studies analysing the miRNA expression signatures in BC, their correlates with specific molecular subtypes and their potential clinical relevance have been reported [8-11].

The miR-200 family of miRNAs consists of five members grouped in two independent transcriptional clusters: miR-200a, 200b and 429, located on chromosome 1p36; and miR-200c and 141, located on 12p13. Deregulation of miR-200 family of microRNAs in cancer [12,13] has been related to epithelial to-mesenchymal transition and cell-plasticity, apoptotic response, molecular subtype, oestrogen regulation, control of the growth and function of stem cells and regulation of the downstream transcriptional program that mediate distant metastasis. Also, regulatory functions of miR-200 s in tumour angiogenesis have been recently described [14]. However, in vitro and functional studies have yielded conflicting results regarding the net effect of miR-200 s in suppressing or promoting metastasis in different cellular contexts and cancer types [15-17].

MicroRNAs can be detected in the blood and studies indicate they are particularly stable and abundant [18,19]. Circulating miRNAs could be actively secreted from tumour cells, but also from non-malignant cells, including immune cells, either microvesicle-associated or free, in a selective manner [20]. In addition, passive leakage derived of apoptosis or necrosis of cancer cells tissue or chronic inflammation could be the source of microRNA founded in total blood, serum or plasma.

Our previous study has shown miR-200c in the blood can distinguish with significant specificity and sensitivity, patients with gastric cancer from healthy controls and remarkably, increased expression levels of miR-200c in blood were significantly associated with poor progression-free and overall survivals in gastric cancer patients [21].

Only a few studies have directly examined the role of miRNAs in the prognosis in BC, the vast majority of which were conducted analysing miRNA expression in the primary breast tumour (revised in [22]). However, little is known concerning the relationship between the blood miRNA expression profiles with the prognosis in BC patients. We first performed a Phase I preclinical study by means of computational tools for miRNAs profiling. Selected miRNAs were evaluated by RT-qPCR in BC and hematopoietic cell lines, control bloods, and blood from metastatic BC patients. Based on these results miR-141 and mir-200c were chosen for further analysis in BC patients [23].

Hence, we hypothesised that the quantitative detection of the miR-200 family in the whole blood could be useful as clinical biomarker in BC patients. In that sense, the blood miR-200 cluster expression might correlate with BC diagnosis, staging and prognosis. In the present study, we found that miR-200c and miR-141 expression levels were deregulated in the blood of BC patients. Likewise, the blood levels of miR-200c and miR-141 emerged as compelling and independent prognostic signature for the progression and survival of BC patients.

## Methods

### Participants

Consecutive female BC outpatients were included from the medical oncology unit at University hospital in La Coruña, Spain. Inclusion criteria were: Confirmed pathologic diagnosis of invasive BC; stage I–III with no prior systemic therapy; stage IV patients with no previous systemic therapy or in confirmed progression after such treatment; written informed consent.

Exclusion criteria were defined as: previous invasive non-breast cancer; coagulopathies or platelets ***<*** 20,000 × 10^9^/L; any previous systemic therapy for BC except relapsed or stage IV patients with confirmed progressive disease; prior pelvic radiation; previous bisphosphonate therapy.

The diagnostic work-up included clinical examination, blood sampling with CA 15.3 serum determination, mammography, chest x-ray, abdominal ultrasound and bone scan. Computed tomography scanning of the chest, abdomen and pelvis was performed on stage IV patients.

The patients were followed up clinically every 3 months during the first 2 years, every 6 months for 3 years and in a yearly basis afterwards to monitor disease progression. Mammographic evaluation was performed every year during all the follow up period.

The controls (all females) were recruited from the patients’ family and relatives. We only excluded subjects with a previous history of malignant disease. Thus, controls with different chronic but stable diseases (e., hypertension, diabetes mellitus or heart disease) were eligible and consecutively recruited. The control cohort was selected to include an age distribution that was comparable to the patient group.

The peripheral venous blood (PB) for quantitative reverse transcription PCR (RT-qPCR) analysis was collected in EDTA-containing tubes (10 mL). The first 5 mL of collected blood was discarded to avoid contamination with epidermal cells. Then, the PB was further diluted in RNA*later* and frozen at −20°C for storage until RNA extraction

This study was approved by the Ethics Committee of Clinical Investigation of Galicia (Spain) and conducted in compliance with the Helsinki Declaration. Written informed consents were obtained from all the patients and the controls prior to their inclusion in the study.

### Pathological analyses

The primary tumour and axillaries lymph nodes collected during surgery were processed on a routine diagnostic basis. Histological type, tumour size and nodal involvement were analysed, and the disease was staged according to the TNM system [24]. Tumour grading was performed according to modified Bloom–Richardson score. Immunohistochemistry (IHC) was performed for oestrogen receptor (ER), progesterone receptor (PgR), Ki-67antigen (MIB-1) and HER2. Immunopositivity was recorded when 10% (ER, PgR) of the nucleus of tumour cells were stained. HER2 required distinct membranous staining for being considered positive (3+). The HER2 status of tumours with an IHC score of 2+ was determined by the fluorescence *in situ* hybridization results.

Residual disease status at the time of blood sampling was classified as R0 when no residual disease was present after surgery, R1 when microscopic residual disease was found and R2 in the presence of macroscopic disease. The patients from whom the blood was obtained before the start of neoadjuvant treatment were categorised as R2. When surgery was not performed, the pathological diagnosis was based on radiological-guided biopsies.

### Blood microRNA isolation and reverse-transcription quantitative PCR (RT-qPCR)

MiRNA extraction from blood was performed with the RiboPure-Blood Kit (Ambion Inc, Austin, TX). The procedure was performed using 0.5 mL of whole blood. The mirVana^TM^ RT-qPCR miRNA Detection Kit (Ambion Inc, Austin, TX) was used to detect and quantify miRNA expression. To control input variability and sample normalisation, primer sets specific for the small RNA species U6 snRNA (Ambion, AM30303) and 5S rRNA (Ambion, AM30302) were used. Real-time PCR was performed on the LightCycler® 480 Instrument (Roche, Mannheim, Germany).

The Relative Expression Software Tool (REST) was used to analyse the relative miRNA expression in each sample and to determine the fold difference for every miRNA [25]. The expression levels of the target miRNAs were standardised using an index containing 5S rRNA and U6 snRNA.

All the procedures have been described previously [21]. For details, refer to Additional file 1.

MiRNA analyses were performed with no knowledge of the clinical or follow-up data.

### Bioinformatics and microRNAs expression profiling

MiRNA expression data from previously published BC cohorts [9,10] were retrieved. Selected microRNAs, miR-200c and miR-141 were analysed further to assess whether they were associated with clinic and pathologic factors.

The online tool MIRUMIR [26] was used to estimate the power of miR-200c and miR-141 tumour expression to serve as potential biomarkers to predict survival of BC patients. MIRUMIR performs survival analyses across several available data sets. False discovery rate control procedure is implemented to adjust *P*-values for multiple testing. MIRUMIR is freely available at http://www.bioprofiling.de/MIRUMIR.

In addition, the PROGmiR tool [27] available at http://www.compbio.iupui.edu/progmir was also used to study overall survival implications for miR-200c and miR-141 in BC. The BC expression data comes from The Cancer Genome Atlas (TCGA; https://tcga-data.nci.nih.gov/tcga). This dataset include survival data of 727 cases of invasive breast carcinoma. MicroRNA expression data was obtained using the Illumina Genome Analyzer (GA) and HiSeq platforms.

Finally, to more comprehensively profile circulating miR-141 and miR-200c as potential markers of BC, we obtain their expression in serum, plasma or total blood in the genome-wide studies deposited in NCBI’s Gene Expression Omnibus (GEO) [28]. The values of the specific miRNAs were retrieved through of the GEO2R web application, available at http://www.ncbi.nlm.nih.gov/geo/geo2r/.

### Study design and statistical analyses

The primary aims were to estimate the diagnostic accuracy and usefulness of miRNA as measured by RT-qPCR in the blood of BC patients as a clinical biomarker and to determine its potential prognostic value. The study was performed following the proposed guidelines of the Early Detection Research Network [29]. The design and results are presented in accordance with the REMARK [30] and MIQE guidelines [31].

The potential correlation among blood miRNA levels and the clinical and pathological features of the study subjects were analysed. The normality of the distribution of miRNA expression was analysed using the Kolmogorov-Smirnov test. Thus, parametric or non-parametric statistics were used, as appropriate. The relationships between miRNAs levels and the quantitative clinical variables were analysed using the Spearman correlation. The Cutoff Finder software [32] was used for receiver operating characteristic (ROC) curves analysis and miRNAs expression cutoffs determinations. The ROC curves were constructed by plotting sensitivity (Y-axis) vs 1-specificity (X-axis) and the areas under the curve (AUC) were calculated. The method used was based on the maximization of Youden’s J statistics. In this first step, the cutoff is optimized for discriminating controls and BC patients based on miRNAs expression. In the second step, the Cutoff Finder tool fits Cox proportional hazard models to the dichotomized miRNA expression in the BC cohort and the survival variables (OS and PFS). These prognostic cutoffs are defined as the points with the most significant (log-rank test) split. Hazard ratios (HRs) including 95% confidence intervals are calculated to assess the stability and significance of the dichotomization.

Significances of correlations with overall survival (OS) and progression-free survival (PFS) were determined. PFS was measured as the time between the baseline blood sampling for miRNA analysis and the documentation of first BC progression, based on clinical and radiological findings, second primary tumour or death from any cause (events). OS was measured from the time at which the baseline blood sample was obtained to the date of death from any cause or date of last follow-up. The patients who were alive and progression-free at the time of analysis were censored by using the time between the blood assessment and their most recent follow-up evaluations.

Multivariate survival analyses (PFS and OS) were performed using Cox regression models. All statistical tests were two-sided and *P* values less than 0.05 were considered significant. SPSS Statistics 19.0 for Windows (IBM Corporation, Armonk, NY, USA, 2011) and Graph Pad Prism 5 (GraphPad Software, La Jolla, CA, USA, 2007) were used for data analyses.

The statistical power of the study was estimated *post-hoc*, taking into account a probability of survival at the end of the study of 0.75 in the low-risk miRNA signature group and 0.35 in the poor-prognostic subgroup. The poor-prognostic subgroup was defined by an increased expression of miR-200c and/or down-regulation of miR-141 in the patient’s bloods. With the sample size of 57 patients, the study was able to demonstrate by two-sided log-rank test, a significant difference in OS, with an alpha-error of 0.05 and a statistical power higher than 80%.

## Results

### Patients and clinical data

From November 2006 to May 2008, 57 female patients with histological proven BC were consecutively recruited for this study. The control cohort included 20 cases. The clinical characteristics of the included subjects are shown (Table 1). The mean age was 54.8 years (standard error of the mean [S.E.M.], 3.2; range, 29 to 73 years) in the control group and 55.4 years (S.E.M., 1.7; range, 27 to 83) in the patient group (*t* test, *P =* 0.853).

**Table 1:** **Characteristics of subjects included in the study**

Characteristic	Patients	Controls	
	*n* = 57 (%)	*n* = 20 (%)	
Age (years, mean ± SD)	55.4 ± 12.8	54.8 ± 14.3	0.853*
<55	28 (49)	12 (60)	0.48**
≥55	29 (51)	8 (40)	
Menopause			
Pre-menopausal	24 (42.1)	N/A	
Post-menopausal	33 (57.9)	N/A	
Histology			
Ductal	50 (87.7)		
Lobular	5 (8.8)		
Other	2 (3.5)		
Histological grade			
1	7 (12.3)		
2	25 (43.9)		
3	23 (40.4)		
TNM Stage			
I	14 (24.6)		
II	13 (22.8)		
III	17 (29.8)		
IV	13 (22.8)		
Lymph nodes involved			
No	20 (35.1)		
Yes	37 (64.9)		
Hormonal Receptors			
Positive	42 (73.7)		
Negative	14 (24.6)		
HER2			
Positive	14 (24.6 )		
Negative	42 (73.7 )		
MIB1			
<25%	40 (70.2)		
>25%	14 (24.6)		
Type			
Luminal	32 (56.1)		
HER2	14 (24.6)		
Triple negative	10 (17.5)		
R0	28 (49.1)		
R2	29 (50.9)		

*Abbreviation: ECOG* Eastern Cooperative Oncology Group performance status. Residual Status (R): R0, no residual tumour; R2, macroscopic residual tumour. The number (percentages) of patients with data avalaible is indicated. *Student t-test. **Chi^2^ test.

The blood was obtained after R0 surgery in 28 patients (49.1%). In 29 (50.9%) patients, the blood samples were obtained before neoadjuvant treatment or in the presence of metastatic disease, both of which were categorised as R2 at the time of blood sampling.

All patients were followed until death or study completion. The last date of follow-up for the survivors was April (2013). Disease progression events occurred in 22 patients (38.6%). The mean PFS was 235.3 weeks (95% CI: 203.6 to 267 weeks). There were 10 progressions among stage I–III patients and 12 progressions of metastatic disease. The mean OS was 264.6 weeks (95% CI: 239.2 to 290 weeks) and 19 patients (33.3%) died. The mean (S.E.M.) follow-up time for the patients still alive at the time of the analysis was 298.2 (2.7) weeks (median, 303.7 weeks; 95% CI: 296.6 to 310.8 weeks).

### Expression of miRNA in blood samples

**Figure 1:**
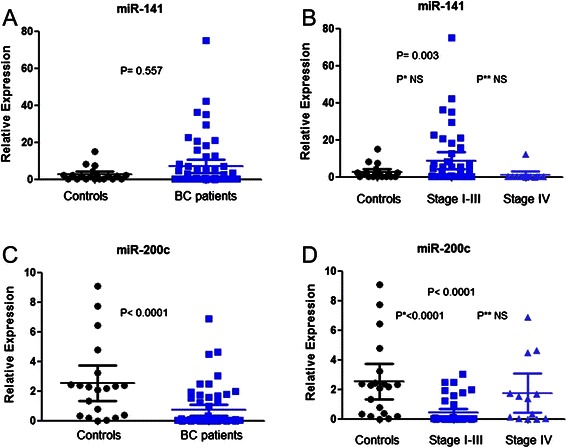
Real time PCR of miR-200c and miR-141 in blood samples. Plots depicting the relative expression for blood levels of miR-141 **(A)** and miR-200c **(C)** between patients and healthy controls, and between stage I-III patients, stage IV patients and healthy controls **(B and D)**. The horizontal bar denotes the mean value for each group. The corresponding *P* values are provided in plots.

Real-time PCR quantitative assessment of miR-141 and miR-200c were performed using 77 blood samples (57 patients and 20 controls). The Figure 1 depicts relative expression for the blood levels of miR-141 and miR-200c.

The blood expression of miR-141 was not significantly (*P =* 0.557) different in patients compared to healthy controls (Figure 1A). The mean relative miR-141 expression (Figure 1B) was 2.615 (S.E.M., 0.83; 95% CI: 0.89 to 4.34) in controls, 8.81 (S.E.M., 2.29; 95% CI: 4.2 to 13.4) in stage I-III patients and 1.06 (S.E.M., 0.93; 95% CI: 0 to 3.09) in stage IV BC patients (*P =* 0.003 Kruskal-Wallis test. Dunnett’s multiple comparison tests: stage I-III *vs* control, *P =* 0.099; stage IV *vs* control, *P =* 0.904). However, the blood levels of miR-141 could not discriminate BC patients from healthy women in ROC analysis.

We compared the expression levels of circulating miR-200c in controls and BC patients. Our data showed miR-200c was downregulated in the blood of BC patients by comparison with its expression in the blood of controls (*P* < 0.0001; Figure 1C). Next, we sought to identify potential differences of the expression levels of miR-200c according to stage. The mean relative miR-200c expression (Figure 1D) was 2.53 (S.E.M., 0.58; 95% CI: 1.31 to 3.74) in controls, 0.41 (S.E.M., 0.13; 95% CI: 0.16 to 0.66) in stage I-III patients and 1.75 (S.E.M., 0.62; 95% CI: 0.41 to 3.09) in stage IV BC patients (*P <* 0.0001; Kruskal-Wallis test. Dunnett’s multiple comparison tests: stage I-III *vs* control, *P <* 0.0001; stage IV *vs* control, *P =* 0.342).

**Figure 2:**
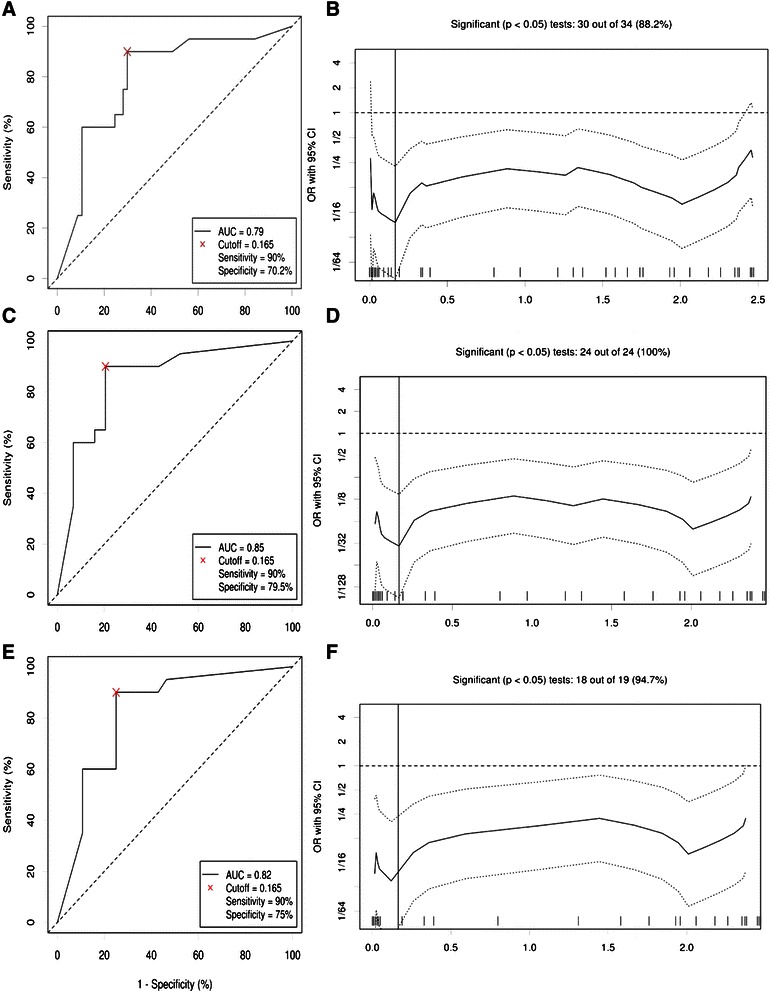
The role of blood miR-200c in breast cancer diagnosis. Receiver-operating characteristic (ROC) curve analysis **(A)** and odds ratio plot **(B)** using blood miR-200c expression levels for discriminating breast cancer patients (n = 57) and healthy controls (n = 20). ROC curves and odds ratio plots for discrimination of stage I-III BC from healthy controls **(Cand D)** and discrimination of stage I-II patients from healthy controls **(E and F)** are also shown.

ROC curve analysis (Figure 2A) showed that the blood levels of miR-200c might serve as negative biomarker for discriminating BC patients from healthy controls, with an AUC (the area under the ROC curve) of 0.79 (95% CI: 0.688 to 0.914; *P <*0.001). At the cut-off value of 0.165, the sensitivity and specificity were 90.0% and 70.2%, respectively. The odds ratio (OR) according to the cut-off value (Figure 2B) was 0.62 (95% CI: 0.45-0.85; *P <* 0.0001).

The ROC curve analysis using blood miR-200c yielded an AUC of 0.850 (95% CI: 0.738 to 0.957; *P <* 0.001; OR: 0.37) in discriminating stage I-III BC from healthy controls as shown in Figure 2C and D. When comparing the relative miR-200c levels in controls and stage I-II patients (Figure 2E and F), the AUC was 0.82 (95% CI: 0.694 to 0.945; *P <* 0.001; OR: 0.45) with a sensitivity of 90%, and a specificity of 75%.

### Clinical and pathological characteristics and miRNA levels in the blood

The clinical and pathological characteristics and the miR-200c and miR-141 expression levels in the blood from BC patients are given (Table 2). The correlations of quantitative clinical and laboratory parameters and miRNAs levels are summarized (Table 3). The miR-200c levels were not related to any of the clinical and pathological characteristics analysed. There was a tendency (*P =* 0.054) to higher levels in the stage IV group compared to stages I-III group. MiR-141 levels were significantly higher in the blood of the patients with lymph node metastasis (*P =* 0.014) and HER2 negative tumours (*P* = 0.037). In stages I to III BC patients, we evaluated the miR-200c and miR-141 levels according to timing of blood sampling (post- vs. pre-operative). The levels of each miRNA in the post- operative vs. pre-operative samples were not significantly different (Table 2). However, the pre-and post-resection samples were not paired from the same patients.

**Table 2:** **Distribution of clinical and pathological parameters and levels of miR-200c and miR-141 in blood**

Parameter	*n*	miR-200c	*P*value	miR-141	*P* value
Age (years)			0.190		0.240
<55	28	0.35 (0.72)		7.71 (12.47)	
≥55	29	1.07 (1.74)		6.4 (15.1)	
Menopause			0.572		0.258
Pre-menopausal	24	0.37 (0.68)		7.8 (12.96)	
Post-menopausal	33	0.97 (1.68)		6.5 (14.5)	
Histology			0.140*		0.712*
Ductal	50	0.69 (1.36)		7.26 (14.22)	
Lobular	5	1.3 (1.95)		10.42 (14.67)	
Other	2	0.01 (0.01)		0.2 (0.02)	
Histological grade			0.106*		0.703*
1	7	1.79 (1.59)		3.84 (4.92)	
2	25	0.36 (0.69)		4.19 (7.61)	
3	23	0.77 (1.74)		11.72 (19.31)	
TNM Stage			0.054		*0.001*
I-III	44	0.41 (0.83)		8.81 (15.16)	
IV	13	1.75 (2.22)		1.06 (3.35)	
Lymph nodes involved			0.216		*0.014*
No	20	1.06 (1.34)		1.53 (3.41)	
Yes	37	0.53 (1.38)		10.02 (16.21)	
Hormonal receptors			0.460		0.887
Positive	42	0.79 (1.52)		5.03 (9.35)	
Negative	14	0.44 (0.86)		13.58 (21.85)	
Oestrogen receptors			0.460		0.887
Positive	42	0.79 (1.52)		5.03 (9.35)	
Negative	14	0.44 (0.86)		13.58 (21.85)	
Progesterone receptors			0.653		0.371
Positive	29	0.59 (1.15)		5.96 (10.53)	
Negative	27	0.82 (1.61)		8.46 (16.84)	
HER2			0.833		*0.037*
Positive	14	0.88 (1.41)		1.24 (2.13)	
Negative	42	0.64 (1.39)		9.14 (15.5)	
MIB1			0.073		0.790
<25%	40	0.90 (1.55)		7.93 (15.24)	
>25%	14	0.19 (0.65)		5.99 (10.26)	
Type			0.809*		0.105*
Luminal	32	0.71 (1.52)		6.21 (10.4)	
HER2	14	0.88 (1.41)		1.24 (2.13)	
Triple negative	10	0.41 (0.86)		18.52 (24.35)	
Residual disease			0.554		0.755
R0	27	0.52 (0.97)		8.69 (17.24)	
R2	30	0.89 (1.66)		5.56 (9.73)	
Blood sampling^^^			0.72		0.128
Before surgery	17	0.24 (0.5)		8.69 (17.24)	
After surgery	27	0.52 (0.97)		8.99 (11.59)	

The miRNAs relative expression levels (REL) are shown in arbitrary units. The data represent the mean (standard deviation). *n* indicates the number of patients with data available. ^^^Timing of blood sampling before or after surgery is indicated for stages I to III patients only. Mann–Whitney test. *Kruskal-Wallis test.

**Table 3:** **Correlations of clinical and laboratory parameters and miRNA levels in blood of breast cancer patients**

	miR-200c		miR-141	
	Spearman’s*Rho*	*P*value	Spearman’s*Rho*	*P* value
**Age**	0.217	0.104	−0.208	0.12
**Serum Ca 15.3**	0.163	0.273	−0.361	0.013
**Neutrophils Count**	−0.202	0.133	0.094	0.485
**MIB1 tumour staining**	0.085	0.538	−0.138	0.314
**miR-141**	−0.311	0.019		

The Spearman order correlation analysis showed that miR-200c expression in the blood of BC patients was inversely correlated with the miR-141 level (r_s_ = −0.311, *P =* 0.019). In the control group however, there was no correlation between miR-141 and miR-200c (r_s_ = 0.006, *P =* 0.98).

### Prognostic significance of miR-200c and miR-141 levels in the blood

The HRs for PFS and OS were first estimated considering the actual values of every miRNA as a continuous variable in a Cox regression model. Increasing values for miR-200c were associated with PFS events (HR 1.37; 95% CI: 1.09-1.71; *P =* 0.007) and reduced OS (HR 1.38; 95% CI: 1.11-1.71; *P =* 0.003). In contrast, the miR-141 levels as a continuous variable were not significantly associated with outcomes (HR for PFS, 0.987; 95% CI: 0.95-1.025; *P =* 0.498. HR for OS, 0.986; 95% CI: 0.942-1.032; *P =* 0.542).

To generate survival curves, we converted continuous miRNAs expression values to dichotomous variables, using the Cutoff finder software [32]. This procedure enabled division of samples into classes with high and low expression of microRNA.

Using this approach, miR-141 was down-regulate in the blood of 26.3% (15/57) of the patients. The percentage of patients with miR-141 down-regulation was associated with TNM stage: 18.2% (8/44) in stage I-III patients and 53.8% (7/13) in stage IV patients (Fisher’s exact test, *P =* 0.027). In contrast, high expression of miR-200c was found in 24.6% of the patients (14/57). This overexpression was also associated with stage IV (53.8% of the patients; Fisher’s exact test, *P =* 0.01).

**Figure 3:**
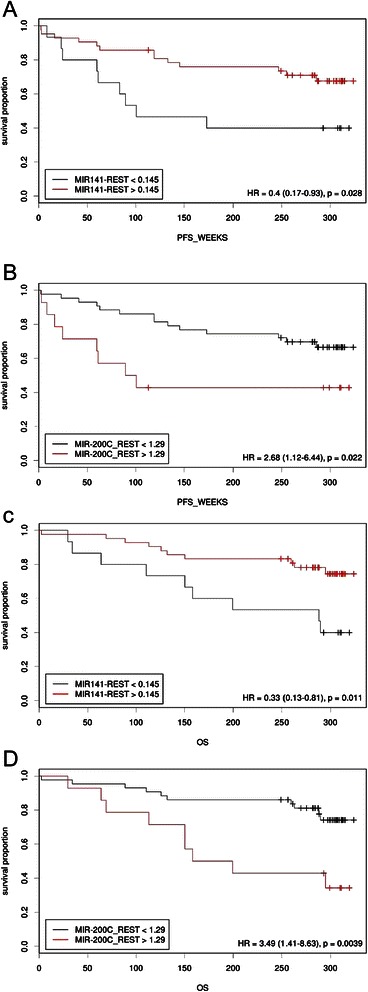
miR-200c and miR-141 expression levels measured in peripheral blood are associated with poor prognosis in breast cancer patients. Kaplan-Meier curves showing **(A and B)** the progression-free survival (PFS) and **(C and D)** the overall survival (OS) of 57 breast cancer patients with high or low blood expression levels of microRNA. Continuous miRNA expression levels measured using RT-qPCR were converted to dichotomous variables using the Cutoff software (see text). The *P* values were computed using the Log-rank test.

**Figure 4:**
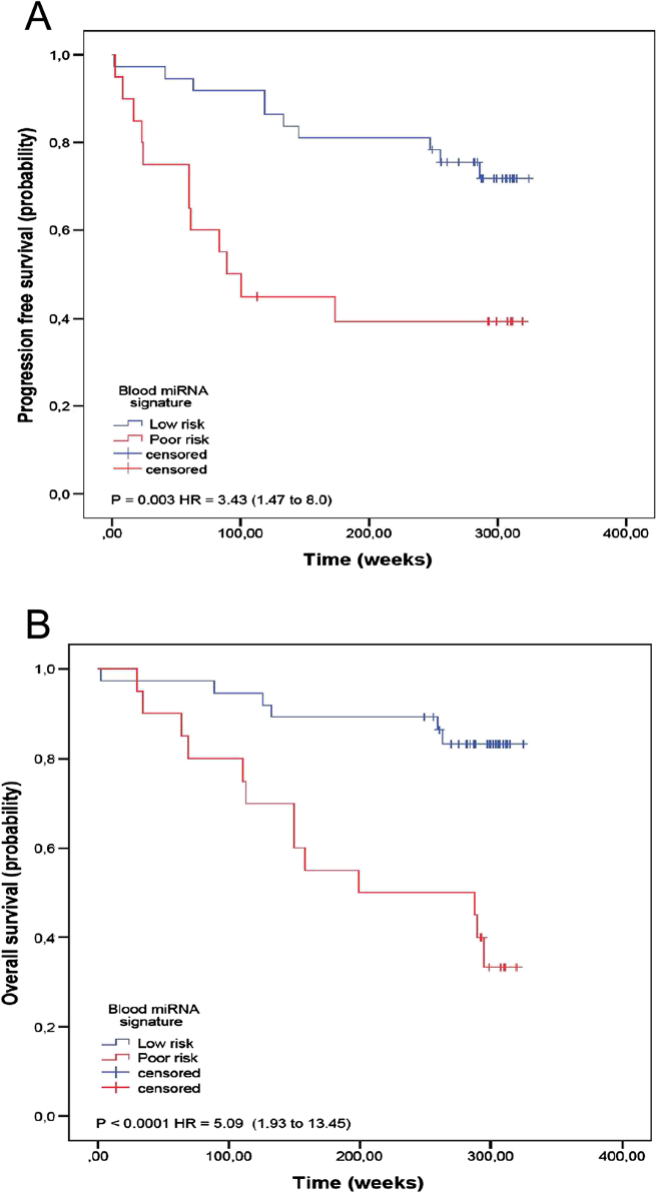
Poor prognostic blood miRNA signature. Kaplan-Meier analysis and log-rank test showed that patients with higher levels of blood miR-200c and/or low levels of miR-141 had significantly poorer progression-free survival **(A)** and overall survival **(B)**.

**Figure 5:**
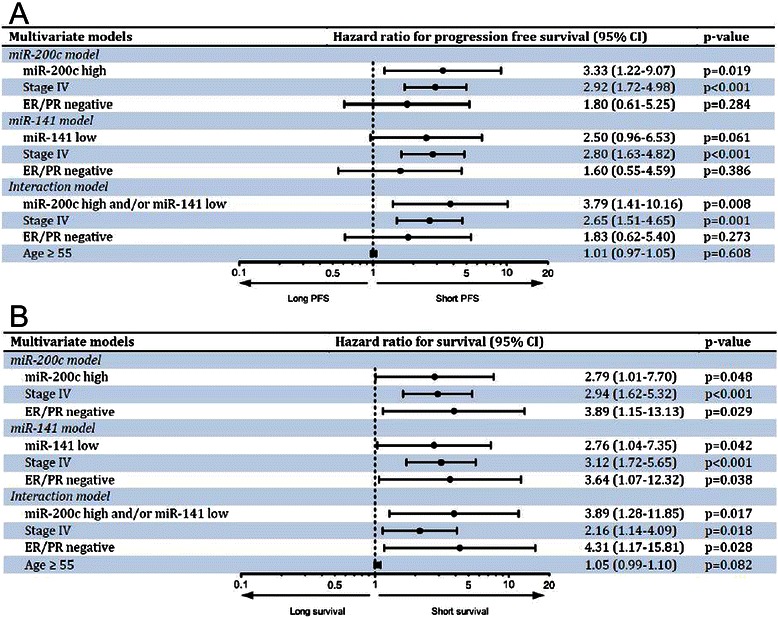
Blood miR-200c and/or miR-141 as prognostic factors in patients with breast cancer. Multivariate models showing the ability of high miR-200c levels, low miR-141 levels, and the combination of both as prognostic factors for predicting progression free survival **(A)** and overall survival **(B)** in breast cancer patients.

The Kaplan-Meier analysis and the log-rank test were used to calculate the effect of miR-200c and miR-141 blood expression on patient survival (Figures 3 and 4). Specifically, the mean overall survival and progression-free survival time of patients whose bloods expressed high levels of miR-200c (>1.29 relative expression value) was 201.48 weeks (median, 158.29 weeks) and 162.84 weeks (median, 89.43 weeks) respectively, whereas the mean OS and PFS time of those with low levels of miR-200c expression was 284.7 weeks (log-rank *P =*0.004) and 258.85 weeks (long-rank *P =*0.022), respectively (Figure 3B and D). The median was not reached in the low miR-200c subgroup. A significant association between a high miR-200c blood level and poor PFS (HR 3.33; 95% CI: 1.22 to 9.07; *P =* 0.019) and OS (HR 2.79; 95% CI: 1.01 to 7.7); *P =* 0.048) was found, with independence of tumour stage and hormonal receptors status as depicted (Figure 5A).

Moreover, low expression levels of miR-141 (<0.145 relative expression value) in BC patient bloods (Figure 3A and C) were found to be associated with poorer progression-free survival time (mean: 169.37 versus 258.12 weeks; log-rank *P =* 0.028) and overall survival time (mean: 216.01 versus 281.9 weeks; log-rank *P =* 0.011). The median was not reached in the high miR-141 subgroup. A decreased miR-141 level was an indicator of a poor prognosis (HR for death, 2.76; 95% CI: 1.04 to 7.35; *P =* 0.042) independently of stage and hormonal receptors. The association of low miR-141 level with progression events when adjusted for stage and hormonal receptors, however, did not reach the statistical significance (Figure 5A; HR, 2.50; 95% CI: 0.96 to 6.53; *P =* 0.061).

To further evaluate whether blood miR-200 s deregulation can predict BC prognosis, we next performed survival analysis. Kaplan-Meier analysis showed that patients with higher levels of blood miR-200c and /or low levels of miR-141 had significantly poorer progression-free survival (*P* = 0.003; log-rank test; Figure 4A) and overall survival (*P* < 0.0001; log-rank test; Figure 4B). The results of the Cox proportional hazards model incorporating a “poor prognostic” blood miRNA signature are shown (Figure 5B). Multivariate analyses included age, tumour stage, hormonal receptors and microRNA levels. When paired in an interaction model, high miR-200c and/or low miR-141 levels had a greater association with decreased survival (HR, 3.89; 95% CI: 1.28 to 11.85; *P =*0.017) and shorter PFS (HR, 3.79; 95% CI: 1.41 to 10.16; *P =*0.008) than either one alone.

### Bioinformatics and microRNAs expression profiling

The miR-200c and miR-141 expression levels by oligonucleotide microarray profiling of a panel of 20 BC samples were retrieved from Mattie et al. [9]. This series included three common phenotypes (9/20, ErbB2-positive/ER-negative; 4/20, ErbB2-positive/ER-positive; 7/20, ErbB2-negative/ER-positive). The tumour miR-141 and miR-200c expressions were not associated with the patient age, hormonal receptors, HER2 overexpression, grade, proliferation index, or p53 mutational status.

The associations between miR-200c and miR-141 tumour expression, molecular subtypes and clinic and pathological factors were assessed using the microRNA expression data (GEO accession number GSE7842) provided by Blenkiron et al. [10], including 93 primary breast tumour samples. For multiple comparisons, *P* value was adjusted at 0.01. No significant associations between miR-200c and miR-141 with tumour characteristics such as molecular subtype, grade, stage, vascular invasion, ER status, Nottingham Prognostic Index (NPI) as well as TP53 status and HER2 overexpression were found.

Two different datasets, which provide miRNAs expression data and clinical outcomes for BC patients, were identified by MIRUMR online tool [26]. In the first dataset, (accession number GSE37405) low miR-141 tumour expression (*P*-values corrected by FDR, 0.03308) and low miR-200c tumour expression (*P*-values corrected by FDR, 0.02324) were associated with a reduced overall survival in high-risk oestrogen receptor positive BC patients (Additional files 2 and 3). By contrast, in the second dataset (GEO accession number GSE22216) that included 189 early primary BC patients, no survival differences were found according to miR-141 (*P =* 0.486) and miR-200c (*P =* 0.469) tumour expression (Additional files 4 and 5).

We also used the PROGmiR tool [26] to create Kaplan-Meier survival plots for miR-200c and miR-141 using the BC TCGA data. Overall survival at 3 and 5 years were not significantly different according tumour levels of miR-200c and miR-141. However, with a longer follow-up, the survival times became significantly better in the high microRNA expressions groups. The hazard ratio and *P* values for the proportional hazards model are also given (Additional files 6, 7, 8 and 9).

The data about circulating miR-141 and miR-200c expression in three genome-wide studies deposited in NCBI’s Gene Expression Omnibus (GEO) were retrieved and analysed [33-35]. These studies included plasma (two studies) or total blood (one study) of control healthy women and early BC patients. The characteristics and results of these studies are depicted (Additional file 10). The levels of miR-141 and miR-200c in the plasma were not significantly different between early BC and controls. However, miR-141 was lower in total blood of the BC cohort in comparison to controls (*P =* 0.029). There was a trend to a negative correlation between circulating miR-141 and miR-200c expression.

## Discussion

Blood biomarkers that provide accurate diagnostic and prognostic information for women with BC are urgently required. MicroRNAs are deregulated in BC and histological and molecular subtypes are characterised by specific microRNA profiles. The deregulated expression of miRNAs in both tumour tissues and the blood compartment has led to the search for miRNAs to predict presence of cancer and indicate its overall prognosis [8-11]. To date, most of the studies in BC have focused on the potential role of circulating (plasma or serum) miRNAs as biomarkers for diagnosis and detection of early disease and most of them are based on the testing of multiple miRNAs, using high-throughput technologies [19,33-35]. However, very few studies have explored the capabilities of the blood miRNA expression in predicting the clinical outcome of BC patients.

We hypothesize the deregulated expression of circulating and cellular miRNAs present in the whole blood can identify the presence of BC, and could thus be developed into a prognostic signature. Our study did not pursue the current tendency to examine circulating miRNAs in plasma or serum using high-throughput technologies. In contrast it is focused on the selective testing of two members of the miR-200 family of microRNAs, miR-200c and miR-141, in the whole blood. Although the feasibility of using miRNA expression profile in whole blood as the basis for recognition of several diseases has been demonstrated [18] its potential prognostic value in cancer has not been comprehensively explored.

We found that miR-200c/miR-141 expression in the blood of BC patients is deregulated comparing with controls and, furthermore miR-200c and miR-141 levels were associated with distinct disease-free survival and overall survival of patients. Both of the univariate and multivariate analyses indicated that miR-200c and miR-141 blood levels were independent prognostic factors for BC outcomes.

Our study showed miR-200c in blood was down-regulated in stages I-III BC patients compared to age-matched controls, discriminating these subsets with an AUC-ROC of 0.85, and compared to patients with metastatic disease. In contrast, a tendency to higher levels of miR-141 in the blood of stage I-III BC patients in comparison with controls and stage IV patients was found. MiR-200c and miR-141 were inversely correlated in the blood of BC patients. Since these miRNAs measurements could discriminate metastatic from early stage BC patients and were associated with prognosis, miR-200c/miR-141 blood levels may represent a BC-specific deregulation with potential functional consequences. Indeed, the blood levels of miR-200c and miR-141 seem to mirror the suggested biphasic role of this family of microRNA during metastatic process [15,16].

In our BC cohort, neither miR-200c nor miR-141 circulating levels were significantly associated with age, menopausal status, histological subtype, tumour grade, hormonal receptors or IHC-based subtypes. The miR-200c levels were numerically higher in stage IV and tumours with lower MIB-1 staining. The miR-141 levels were lower in stage IV, lymph node negative patients and HER2 negative tumours. To validate these results, we used previously reported data on miRNAs profiling studies in BC. Similar to our findings, miR-200c and miR-141 were not associated with molecular subtypes or clinic and pathologic factors analysed [9,10].

One of the strengths of our study is the capability of the whole-blood miR-200 and miR-141 deregulation to predict PFS and OS was interrogated across a set of BC patients with a comprehensive clinical, pathological and long-term outcome data. Even with a relatively low sample size and few events in our patient population we were able to demonstrate the correlation of these miRNAs to PFS and OS. MiR-200c was the most accurate miRNA individually for predicting PFS and OS, and its prediction accuracy increased by a small margin when used in combination with miR-141. The poor-prognostic profile defined by a high miR-200c and/or low miR-141 in the blood levels had a greater association with decreased survival and shorter PFS than either one alone, and it was independent of age, tumour stage and hormonal receptors status in the multivariate Cox’s model.

The sources of miRNAs in the blood are intriguing and whether deregulation in circulating blood miRNAs reflected similar changes in breast tumour tissues is controversial. In that sense, it was surprising to detect reduced concentrations of circulating miR-200c and miR-141 in the whole blood of subsets of our BC patient cohort comparing to age-matched healthy females. Recently Dvinge et al. [17] have demonstrated a global decrease in miRNA expression in breast tumours and described that polycistronic miRNAs can show dependent, independent or even opposite expression patterns in BC. Distinct patterns of miRNAs in circulation and BC tissue had been reported both in murine BC models [36] and clinical series [37].

Furthermore, a recent report suggests that normal and malignant mammary epithelial cells release miRNA into blood and fluids in a specific manner [20]. Microarray and quantitative PCR analyses had indicated the breast tumour cells selectively retain miR-141. In comparison, miR-200c was highly released from cells. The low levels of any particular miRNA in blood could also be caused by an altered RNA polymerase activity or deregulated processing and exporting factors. Therefore, the extracellular accumulation of mature miRNAs is regulated at levels other than the primary transcript abundance in the tumour cells. Roth et al. [38] had found a very low expression of miR-141 in serum from BC patients and healthy females. Moreover the relative yields of miR-141 in serum did not differ significantly between healthy women and women with BC or between M0 and M1 patients. The one previous study that analyse miRNAs in the whole blood in BC patients [34] have included only early stages. They showed a down-regulation of miR-141 in the blood of BC patients while miR-200c was no differentially expressed.

The analysis of miRNA obtained from whole-blood may be advantageous in comparison with serum or plasma determinations, detecting not only those miRNA derived from blood cells comprising circulating tumour cells, but also those secreted in sub-cellular particles such as exosomes or associated with RNA binding proteins and derived from diverse cells and tissues. Compared to serum or plasma, whole blood is easier to collect and has more RNA content, which facilitates reliable and accurate global microRNA expression measurements using less clinical material. Another one of the crucial problems is the efficient and reproducible extraction of small amounts of miRNA from plasma or serum. Therefore, higher yields of miRNAs had been consistently obtained from whole blood samples compared with matched serum or plasma and lower quantification cycles occurred in whole blood compared with matched serum and plasma samples in RT-qPCR experiments [39].

One possibility is that circulating miRNAs are indicative of CTCs and/or metastases. Supporting this concept, Madhavan et al. [40] recently demonstrated that plasmatic levels of miR-200 family members are surrogate markers for CTCs in heavily treated metastatic BC patients and correlate with disease progression and overall survival. However, contradictory results have been described. Roth et al. [38] did not observe any tendency of higher miR-141 levels in serum of CTC-positive BC patients. Sieuwerts et al. [41] found a significant decreased miR-200c transcript levels in the Ep-CAM^+^ circulating tumour cells of metastatic BC patients compared with samples from healthy donors. In contrast, mR-141 transcript levels were not differentially expressed. We hypothesised that changes in miR-200c/miR-141 blood transcripts could reflect at least in part, the presence of tumour cells that have undergone or are undergoing epithelial-mesenchymal (EMT) and mesenchymal-epithelial transitions (MET), a dynamic process likely to be important for efficient metastatic colonisation [42]. These previous reports and our results underline the complex relationships between disease and changes in miRNA expression patterns in blood. Furthermore, the contribution of systemic inflammatory, immunomodulatory or proangiogenic processes to the whole blood microRNA profile cannot be ruled out. Maertzdorf et al. [43] found that blood miR-200c and miR-141 expression levels are reduced in chronic inflammatory conditions. In that sense, deregulation of miRNAs in the blood of BC patients could be related, at least in part, to the host immune and inflammatory context in response to BC.

It has been increasingly recognized that miR-200 family of microRNAs plays an important role in the proliferation, invasiveness and migratory properties of BC cells in cell lines [6,11-13] and experimental models; however, a systematic investigation of how miR-200 s deregulation affects the clinical outcome of BC patients has been poorly defined.

In fact, the relative expression of the miR-200 family in BC compared with normal breast tissue and even though profiling data from primary and metastatic BC samples have showed inconsistent results. Some authors [44] have described the upregulation of miR-200c and miR-141 during the transition from normal mammary epithelia to atypical ductal hyperplasia, and maintained their high expression profiles during later stages of invasive ductal carcinoma. The miR-200 family of microRNAs is differentially down-regulated in metastatic lymph node metastasis compared to paired primary tumour in BCs [45]. However, miR-200 expression was found greater in metastases derived from BC than in primary tumours [16,46].

Overexpression of miR-200 s in primary tumour has been associated with an increased risk of metastasis and poor prognosis (in terms of metastasis-free survival) particularly in ER-positive breast cancers [16]. In contrast, the bioinformatics analysis using MIRUMIR and PROGmiR tools indicate an association between lower levels of miR-200c and miR-141 in breast tumours and reduced overall survival.

Although our preliminary results are promising, several limitations in this study are addressed: (i) as the sample size is still small, further validations in large cohorts and in different ethnic groups are recommended; (ii) a remarkable limitation to this and other studies in this field is the lack of standardized procedures. Different pre-analytical and analytical factors affected the quantification of circulating miRNA, including substrate choice (whole blood, antibody-selected cells, plasma or serum), stabilization reagents, centrifugation or filtration to isolate plasma or serum, miRNA extraction procedures, selection of endogenous internal controls, assay choice, individual variation, and the effect of haemolysis. Because miRNAs are present at lower concentrations in plasma and serum than those found in whole blood, all of these variables could increase the assays variability and the stochastic effects when we quantified any microRNA in serum or plasma samples comparing to whole blood. Currently, there are no consistent reference genes suitable for normalizing circulating microRNA expression. Thus, the selection of references to normalize miRNA levels is still rather empirical. A combination of miRNAs for normalization augments the reliability of the data produced, and has been advocated by different studies. In that sense, we used a combination of U6 and 5S as reference genes.

Finally, the clinical utility of any proposed biomarker might be confirmed and validated in independent studies. In that sense our results regarding the prognostic value of circulating miR-200c deregulation in BC are in line with previous results including ours in gastric cancer and the recently reported works in oesophageal and colorectal cancers [21,40,47,48].

In summary, the results of our pilot study indicate that miR-200c and miR-141 levels are deregulated in the blood of BC patients. Based on the differences between cases and healthy controls, the blood miR-200c assay holds promise as a detection marker in BC. Moreover, we were able to verify that miR-200c and miR-141 in whole blood are promising biomarkers of PFS and OS, both independently and in combination. These results will have to be further verified in large study cohorts that include the different stages and molecular subtypes of BC with adequate follow-up. A special attention to technical challenges and standardization must be pursued in the next validation studies. Furthermore, these findings might have relevant implications for other epithelial cancers where the miR-200 s family of microRNA is also deregulated, widening this exciting and growing field.

## Conclusions

Breast cancer is the leading cause of cancer death in women worldwide. Blood-borne metastases contribute to the great majority of deaths. The discovery of specific biomarkers characterizing the metastatic phenotype holds the promises of personalised therapy and improved prognosis prediction. MicroRNAs can be detected in the blood and studies indicate they are particularly stable and abundant.

We hypothesised that the reverse-transcription quantitative PCR detection of miR-200c and miR-141 in the whole blood could be useful as clinical biomarker in breast cancer patients.

Our results indicate that miR-200c and miR-141 levels are deregulated in the blood of breast cancer patients. Based on the differences between cases and controls, the blood miR-200c assay holds promise as a diagnostic marker. Moreover, miR-200c and miR-141 in whole blood are promising biomarkers of progression-free and overall survival, both independently and in combination.

## Additional files

Additional file 1:
**Supplemental methods and results.**

Additional file 2: **Survival analysis performed with MIRUMIR tool of data from GSE37405 microarray for miR-141 tumour expression.** The *P* value is included in the figure.
Additional file 3: **Survival analysis performed with MIRUMIR tool of data from GSE37405 microarray for miR-200c tumour expression.** The *P* value is included in the figure.
Additional file 4: **Survival analysis performed with MIRUMIR tool of data from GSE22216 microarray for miR-141 tumour expression.** The *P* value is included in the figure.
Additional file 5: **Survival analysis performed with MIRUMIR tool of data from GSE22216 microarray for miR-200c tumour expression.** The *P* value is included in the figure.
Additional file 6: **Survival analysis performed with PROGMIR tool for miR-141 tumour expression values extracted from TCGA breast cancer dataset (3 and 5 years follow-up).** The hazard ratio and *P* value are included in the figure.
Additional file 7: **Survival analysis performed with PROGMIR tool for miR-141 tumour expression values extracted from TCGA breast cancer dataset (>5 years follow-up).** The hazard ratio and *P* value are included in the figure.
Additional file 8: **Survival analysis performed with PROGMIR tool for miR-200c tumour expression values extracted from TCGA breast cancer dataset (3 and 5 years follow-up).** The hazard ratio and *P* value are included in the figure.
Additional file 9: **Survival analysis performed with PROGMIR tool for miR-200c tumour expression values from TCGA breast cancer dataset (>5 years follow-up).** The hazard ratio and *P* value are included in the figure.
Additional file 10:
**Comparative of miR-141 and miR-200c expression in genome-wide circulating miRNA profiling studies in early breast cancer patients.**

## Abbreviations

BC: Breast cancer
CTC: Circulating tumour cells
CNA: Circulating nucleic acids
EMT: Epithelial-to-mesenchymal transition
PB: Peripheral venous blood
RT-qPCR: Reverse transcription quantitative PCR
Cq: Quantification cycle
REST: Relative Expression Software Tool
ROC: Eeceiver operating characteristic curve
AUC: Area under the curve
PFS: Progression-free survival
OS: Overall survival
S.E.M.: Standard error of the mean
ECOG: Eastern Cooperative Oncology Group
