# Supplementary material for: Circulating miR-200c and miR-141 and outcomes in patients with breast cancer
Source: BMC Cancer. 2015 Apr 2;15:297. doi: 10.1186/s12885-015-1238-5 (PMC4405843; doi:10.1186/s12885-015-1238-5)
Supplement: Additional file 1: — Supplemental methods and results. [file 12885_2015_1238_MOESM1_ESM.pdf]

Additional file 1. Supplemental methods and results.

Supplemental methods

Blood microRNA isolation and qRT-PCR

To isolate the miRNA fraction, the RiboPure-Blood Kit was used with the alternate protocol: isolation of small RNAs (Applied Biosystems, Foster City, CA, USA). The procedure was performed using 0.5 mL of whole blood per preparation. The absorbances at 260/280 and 260/230 were assessed using a NanoDrop™ 1000 spectrophotometer (Nanodrop Technologies, Wilmington, DE, USA). The purified RNA was further processed using qRT-PCR or stored at -80ºC until use.

Reverse-transcription (RT) PCR was performed with 25 ng (up to 6.6 µL) of total RNA using the mirVanaTM qRT-PCR miRNA Detection Kit (Ambion, AM1558) with 2 µL 5X RT Buffer, 1 µL 1X RT Primer (Ambion, miR-200c, AM30096*; miR-141, AM2052*) and 0.4 µL of ArrayScript Enzyme Mix for a total volume of 10 µL.

For the PCR reaction, 10 µL of RT reaction and PCR Master Mix were used. The PCR Master Mix consisted of 5 µL 5X PCR buffer containing SYBR Green I, 0.2 µL SuperTaq 5 U/µL, 0.5 µL PCR primers and 9.3 µL of nuclease-free water for a total volume of 15 µL. Real-time PCR was performed on the LightCycler® 480 Instrument (Roche, Mannheim, Germany).

To control input variability and sample normalisation, primer sets specific for the small RNA species U6 snRNA (Ambion, AM30303) and 5S rRNA (Ambion, AM30302) were used. These primer sets were used not only as internal controls but also to verify the integrity of the RNA and the reverse transcription reaction.

Any specimen with inadequate U6 snRNA or 5S rRNA expression would be excluded from the study.

For miR-141 and miR-200c, the PCR cycling conditions and analysis were as follows: denaturation at 95ºC for 8 seconds; cycling, 40 cycles of 95ºC for 5 seconds, 60ºC for 5 seconds and 72ºC for 2 seconds; melting curve analysis, 1 cycle at 95ºC for 5 seconds, 55ºC for 1 minute 5 seconds and 95ºC continuous; and finally, cooling at 40ºC for 10 seconds. The conditions were identical for U6 snRNA and 5S rRNA, except the denaturation step was 1 cycle at 95ºC for 6 seconds.

We verified that the amplification of each PCR product was specific using a melting curve analysis. The amplification efficiency was determined for both target and reference genes. Each assay was performed at least in triplicate.

The quantification cycle (Cq) was performed using LightCycler 480 Quantification software (Roche, Mannheim, Germany). For further data analysis, only those miRNAs with a Cq value equal to or below 35, representing detection of one single-molecule template [1] were considered. Positive and negative controls were included in each experiment.

The Relative Expression Software Tool (REST) was used to analyse the relative miRNA expression in each sample and to determine the fold difference for every miRNA. The expression levels of the target miRNAs were standardised using an index containing 5S rRNA and U6 snRNA.

MiRNA analyses were performed with no knowledge of the clinical or follow-up data.

Supplemental results

Online data about tumour expression of miR-141 and miR-200c and prognosis in breast cancer patients

The online tool MIRUMIR was used to perform Kaplan Meier survival analysis. In the first dataset [2], GEO accession number GSE37405, low miR-141 tumour expression (Additional file 2) and low miR-200c tumour expression (Additional file 3) were associated with a reduced overall survival in high-risk oestrogen receptor positive BC patients (*P*-values corrected by FDR, 0.03308) and 0.02324, respectively]. By contrast, in the second dataset [3], GEO accession number GSE22216, that included 189 early primary BC patients, no survival differences were found according to miR-141 (Additional file 4; $P = 0.486$) and miR-200c (Additional file 5; $P = 0.469$) tumour expression.

The PROGmiR tool available at http://www.compbio.iupui.edu/progmir was also used to study overall survival implications for miR-141 and miR-200c in BC. The dataset (https://tcga-data.nci.nih.gov/tcga) include survival data of 727 cases of invasive breast carcinoma. The Kaplan-Meier survival plots for miR-141 and miR-200c are given. Overall survival at 3 and 5 years were not significantly different according tumour levels of miR-141 (Additional file 6) and miR-200c (Additional file 8). However, with a longer follow-up, the survival times became significantly better in the high microRNA expressions groups (Additional files 7 and 9). The hazard ratio and *P* values for the proportional hazards model are also given.

Supplemental references

1. Mestdagh P, Feys T, Bernard N, Guenther S, Chen C, Speleman F, Vandesompele J: **High-throughput stem-loop RT-qPCR miRNA expression profiling using minute amounts of input RNA.** *Nucleic Acids Res* 2008, **36**:e143.

2. Lyng MB, Lænkholm A-V, Søkilde R, Gravgaard KH, Litman T, Ditzel HJ: **Global microRNA expression profiling of high-risk ER+ breast cancers from patients receiving adjuvant tamoxifen mono-therapy: a DBCG study.** *PLoS One* 2012, **7**:e36170.

3. Buffa FM, Camps C, Winchester L, Snell CE, Gee HE, Sheldon H, Taylor M, Harris AL, Ragoussis J: **microRNA-associated progression pathways and potential therapeutic targets identified by integrated mRNA and microRNA expression profiling in breast cancer.** *Cancer Res* 2011, **71**:5635–45.
