# Supplementary figures and images for: Circulating miR-200c and miR-141 and outcomes in patients with breast cancer
Source: BMC Cancer. 2015 Apr 2;15:297. doi: 10.1186/s12885-015-1238-5 (PMC4405843; doi:10.1186/s12885-015-1238-5)

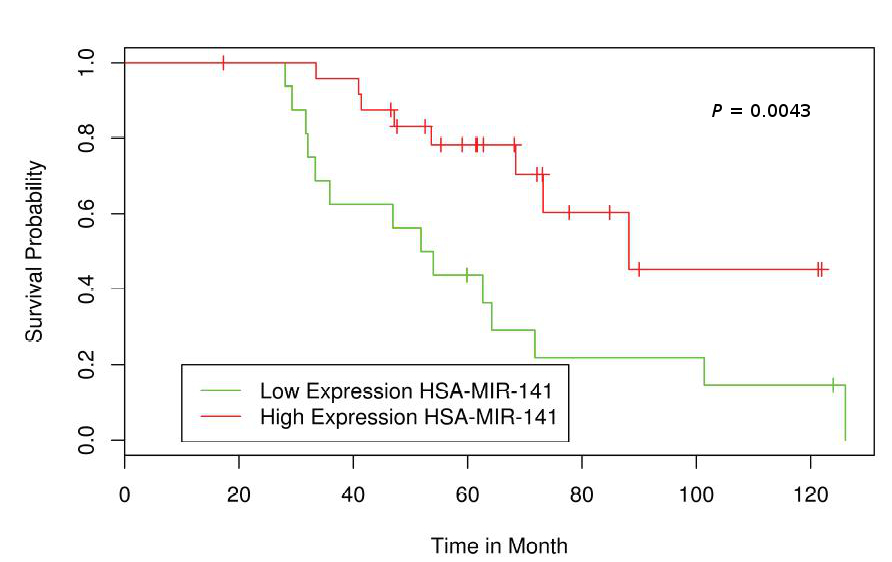

Supplement: Additional file 2: — Survival analysis performed with MIRUMIR tool of data from GSE37405 microarray for miR-141 tumour expression. The P value is included in the figure. [file 12885_2015_1238_MOESM2_ESM.jpeg]

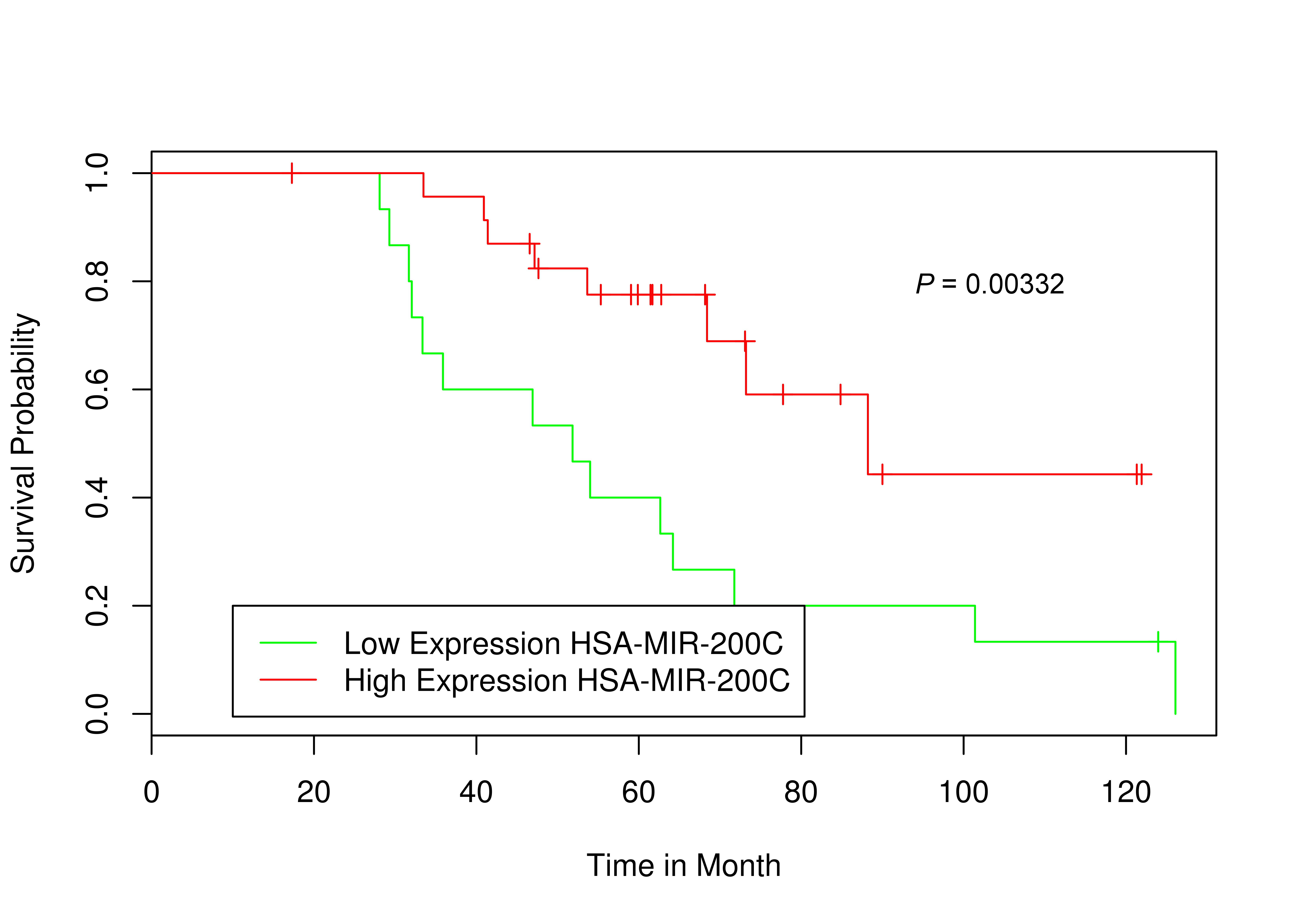

Supplement: Additional file 3: — Survival analysis performed with MIRUMIR tool of data from GSE37405 microarray for miR-200c tumour expression. The P value is included in the figure. [file 12885_2015_1238_MOESM3_ESM.jpeg]

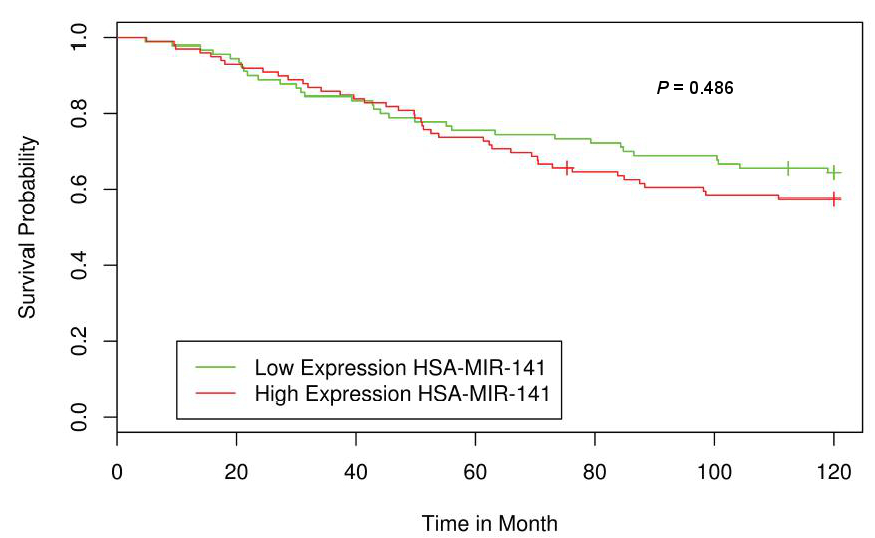

Supplement: Additional file 4: — Survival analysis performed with MIRUMIR tool of data from GSE22216 microarray for miR-141 tumour expression. The P value is included in the figure. [file 12885_2015_1238_MOESM4_ESM.jpeg]

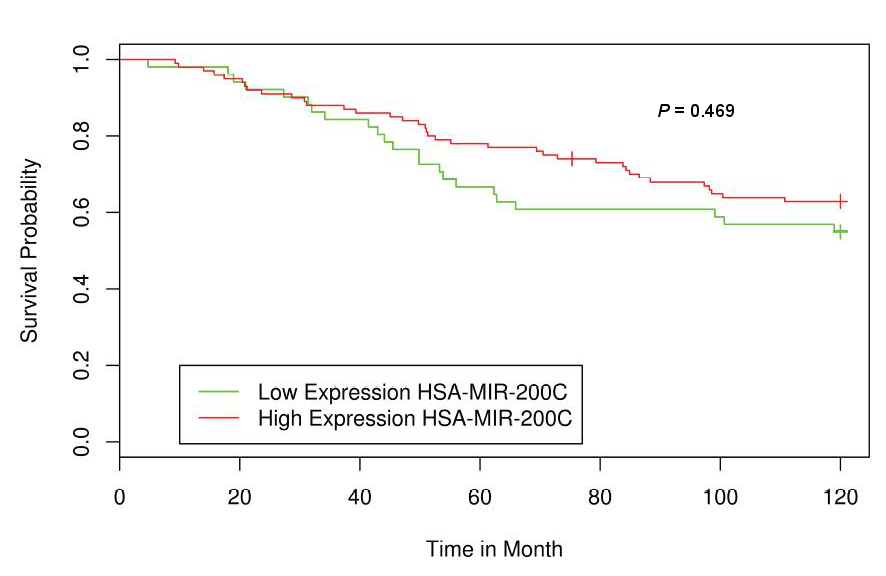

Supplement: Additional file 5: — Survival analysis performed with MIRUMIR tool of data from GSE22216 microarray for miR-200c tumour expression. The P value is included in the figure. [file 12885_2015_1238_MOESM5_ESM.jpeg]

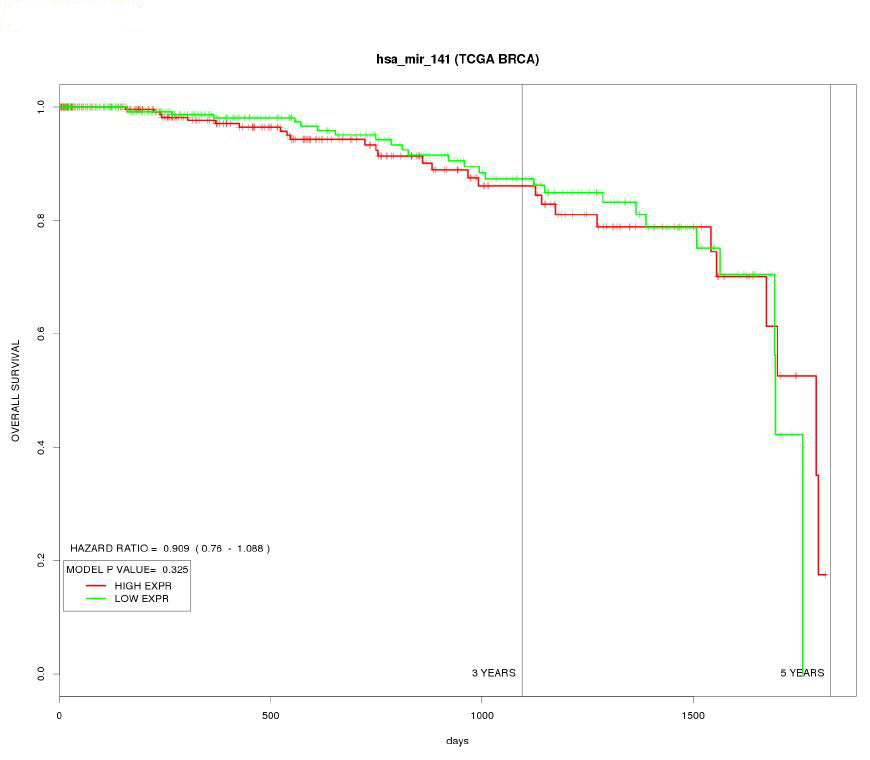

Supplement: Additional file 6: — Survival analysis performed with PROGMIR tool for miR-141 tumour expression values extracted from TCGA breast cancer dataset (3 and 5 years follow-up). The hazard ratio and P value are included in the figure. [file 12885_2015_1238_MOESM6_ESM.jpeg]

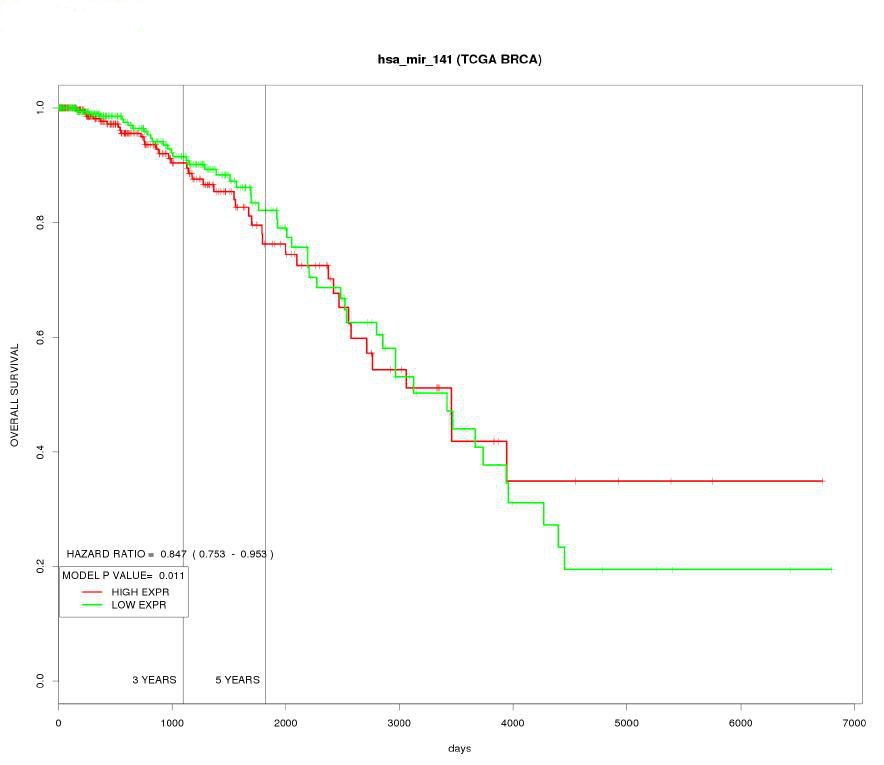

Supplement: Additional file 7: — Survival analysis performed with PROGMIR tool for miR-141 tumour expression values extracted from TCGA breast cancer dataset (>5 years follow-up). The hazard ratio and P value are included in the figure. [file 12885_2015_1238_MOESM7_ESM.jpeg]

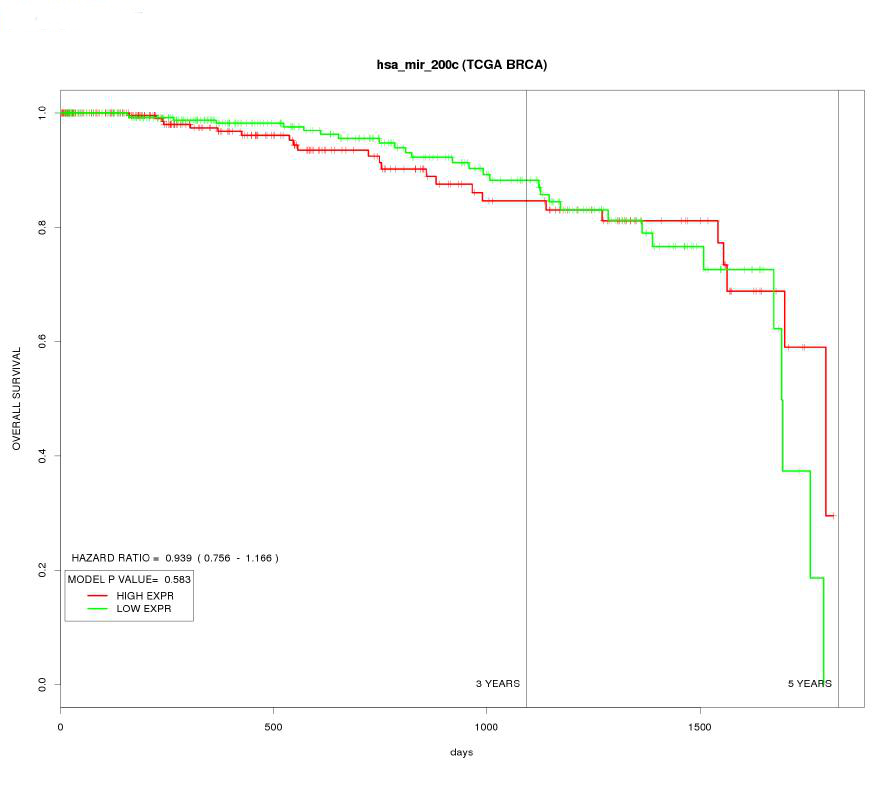

Supplement: Additional file 8: — Survival analysis performed with PROGMIR tool for miR-200c tumour expression values extracted from TCGA breast cancer dataset (3 and 5 years follow-up). The hazard ratio and P value are included in the figure. [file 12885_2015_1238_MOESM8_ESM.jpeg]

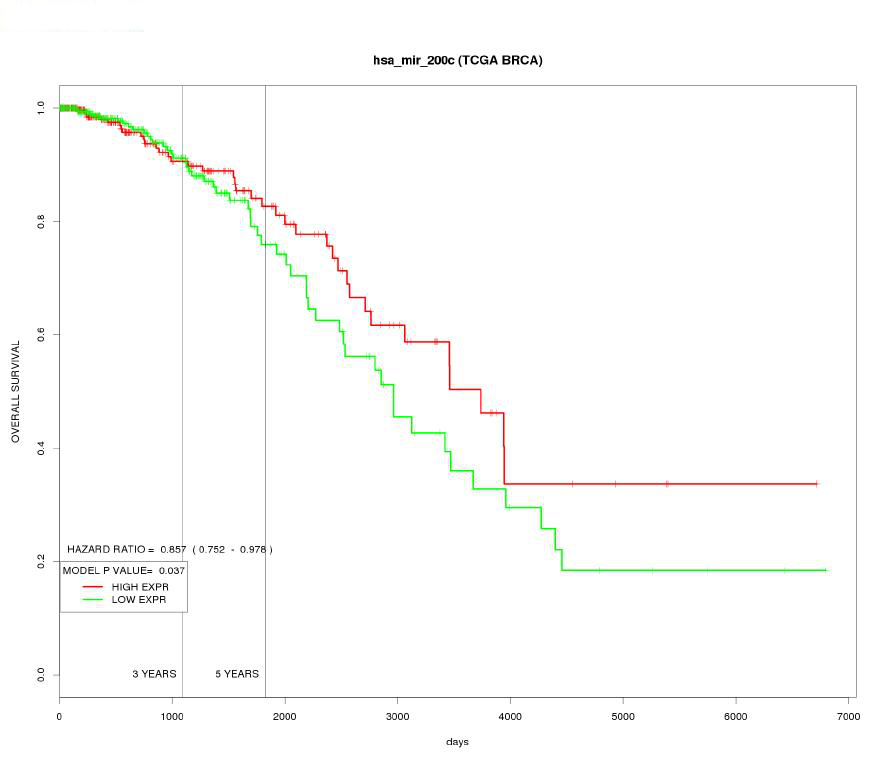

Supplement: Additional file 9: — Survival analysis performed with PROGMIR tool for miR-200c tumour expression values from TCGA breast cancer dataset (>5 years follow-up). The hazard ratio and P value are included in the figure. [file 12885_2015_1238_MOESM9_ESM.jpeg]
